# Supplementary figures and images for: Expansion of lysosomal capacity in early adult neurons driven by TFEB/HLH-30 protects dendrite maintenance during aging in Caenorhabditis elegans
Source: PLoS Biol. 2025 Sep 30;23(9):e3002957. doi: 10.1371/journal.pbio.3002957 (PMC12510649; doi:10.1371/journal.pbio.3002957)

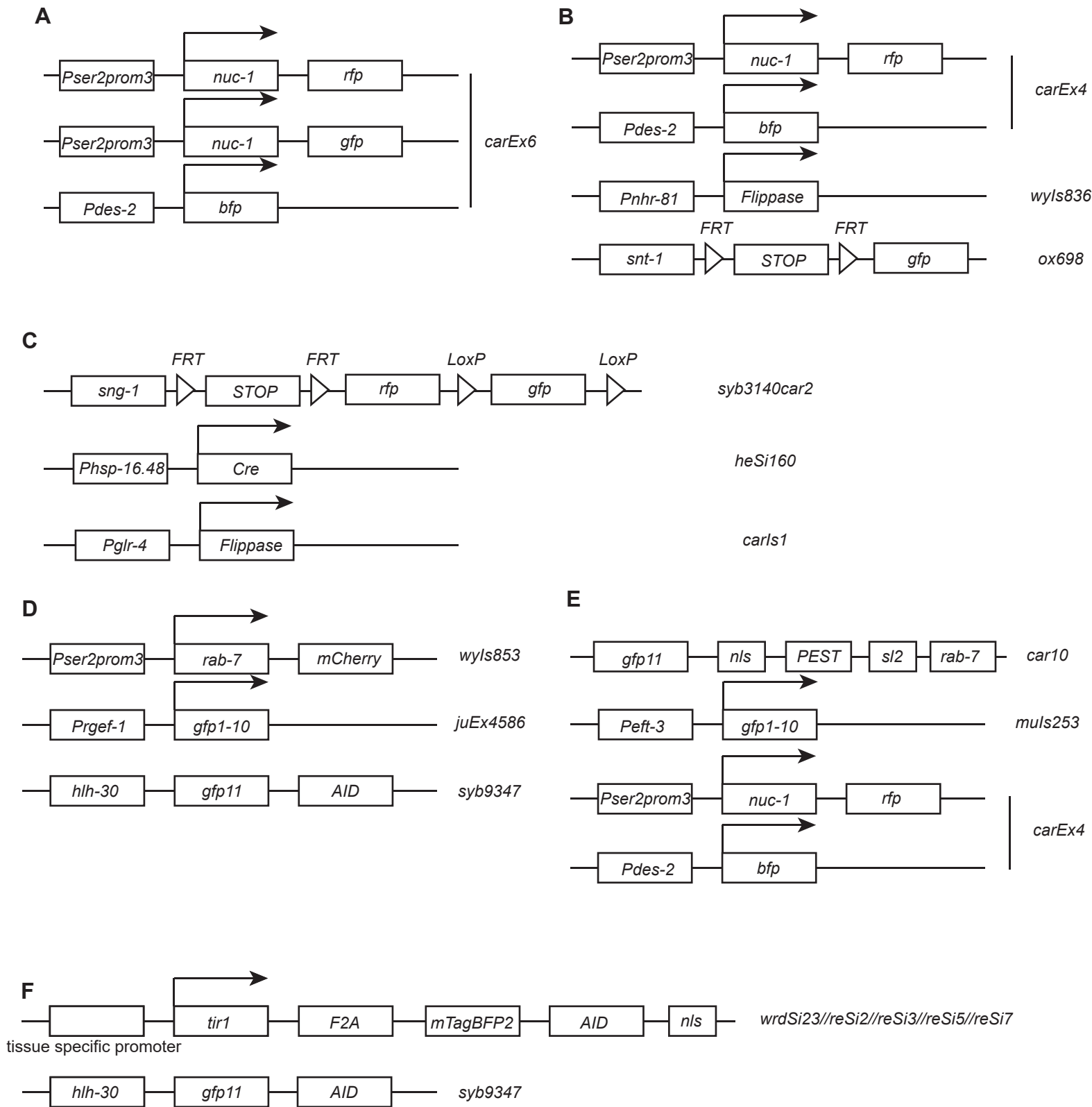

**Fig S1**

Supplement: S1 Fig — (A) Lysosome acidity reporter, composed of three co-injected plasmids. (B) Lysosome degradative capacity reporter. The nuc-1::rfp and bfp morphology marker were co-injected, generating the transgene carEx4. The Pnhr-81> Flippase expresses in seam cells, including the PVD neuron’s grandmother, and was expressed from wyIs836 [50]. The snt-1(ox698) allele is FLP-on SNT-1::GFP [51]. (C) Design for the SNG-1::ARGO-tag [54]. (D) Design for fluorescent reporter of endogenous HLH-30 localization. (E) Design for rab-7 transcriptional reporter, which was inserted into the rab-7 endogenous genomic locus directly after the start codon. (F) Design of the hlh-30::AID allele and tissue-specific TIR1 expression transgenes for the Auxin-inducible degradation (AID) experiments. (PDF) [file pbio.3002957.s001.pdf]

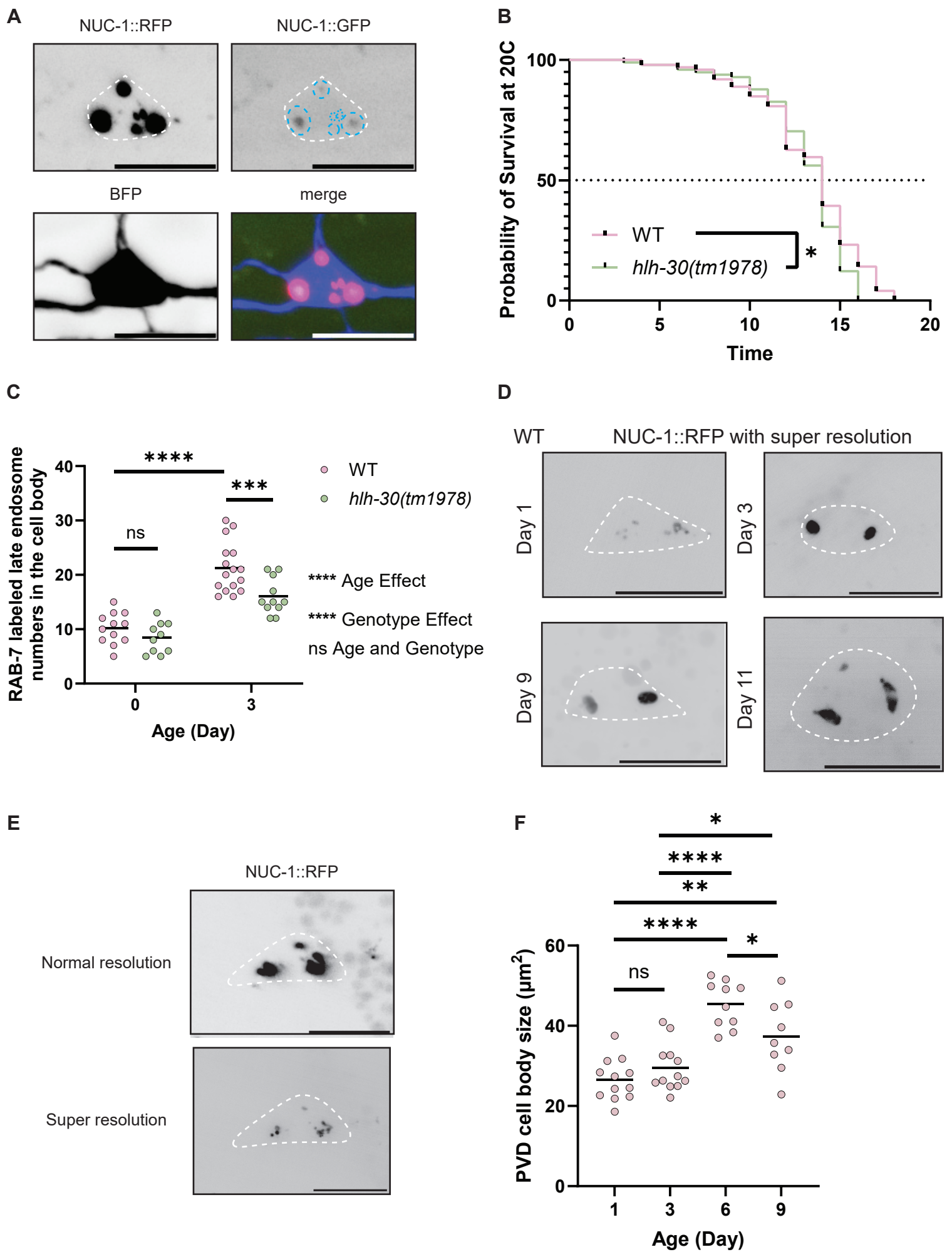

Fig S2

Supplement: S2 Fig — (A) Example images of the NUC-1::RFP/GFP lysosomal acidity reporter in Day 9 adults. White dashed lines indicate neuron cell body and blue dashed lines outline lysosomal compartments. (B) Lifespan analysis of WT vs. the hlh-30 mutant. *P < 0.05, Kaplan–Meier. n = 99 (WT) and 98 (hlh-30 mutant) animals spread across four plates. (C) The hlh-30 mutant has fewer endogenously tagged GFP::RAB-7 endosomes in the PVD neuron cell body at Day 3 of adulthood compared to WT and shows no difference at Day 0. ****P < 0.0001, ***P < 0.001, ns: not significant, Two-way ANOVA with Sidak post-test. (D) Example images of lysosomal compartments in PVD neuron cell body in WT across adulthood. White dashed lines indicate the edge of the cell body. Scale bar = 10 μm. (E) Comparison of the NUC-1::RFP fluorescence from a Day 3 WT adult with standard confocal microscopy versus super resolution. Scale bar = 10 μm. (F) PVD neuron cell body size, quantified using Pdes2>bfp, is similar between Day 1 and Day 3 adults but significantly increases by Day 6 of adulthood. ****P < 0.0001, **P < 0.001, *P < 0.05, ns: not significant. One way ANOVA with Tukey post-test. The data underlying the graphs shown in the figure can be found in S1 Data. (PDF) [file pbio.3002957.s002.pdf]

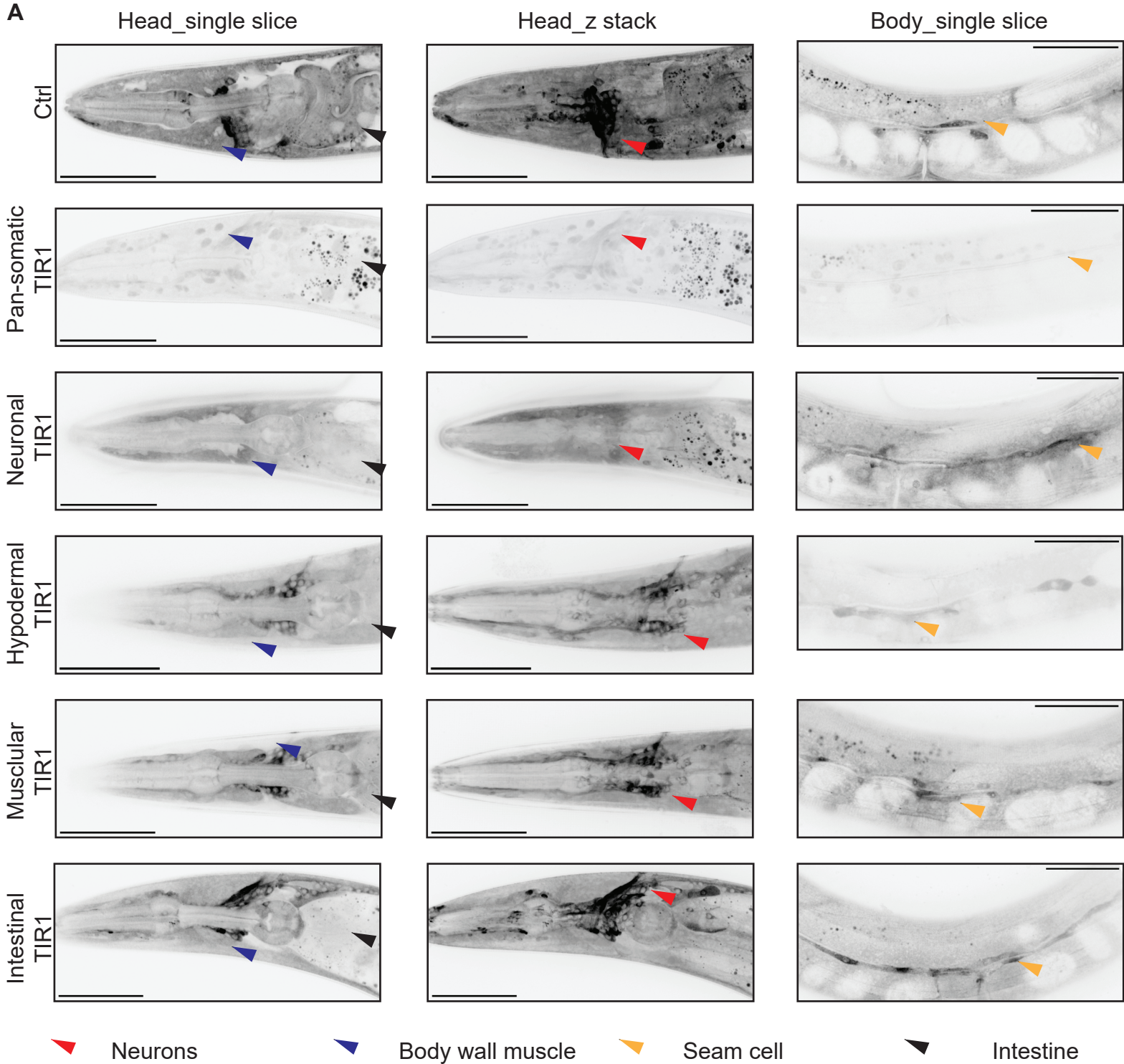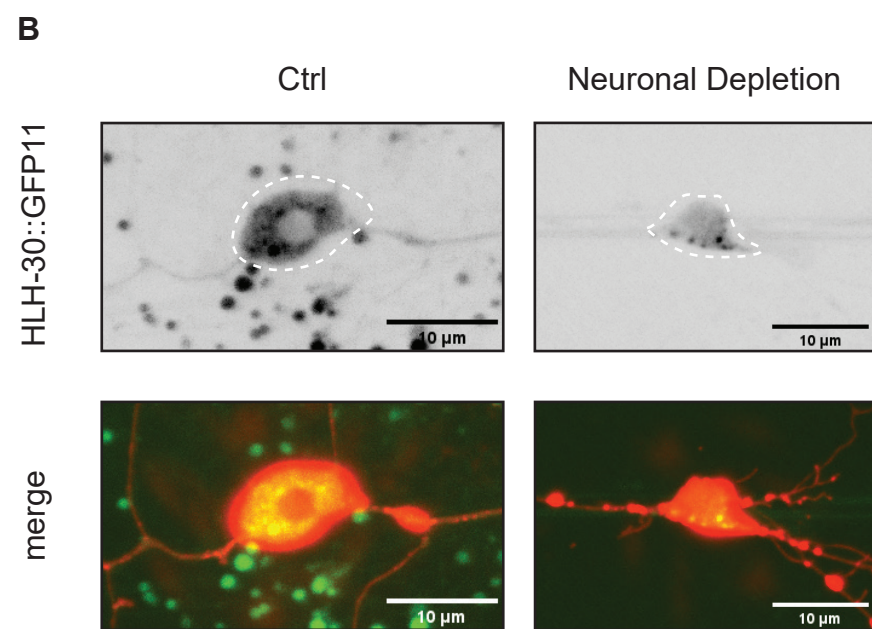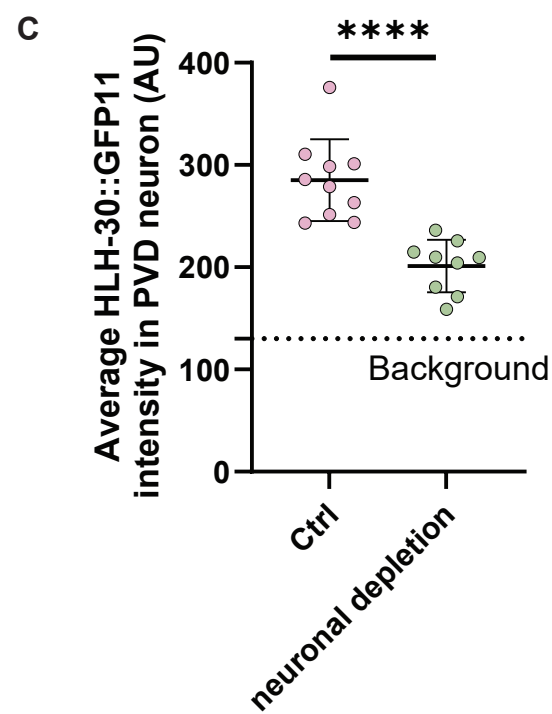

**Fig S3**

Supplement: S3 Fig — (A) Example images showing the effect of tissue-specific TIR1 alleles + auxin on fluorescence of HLH-30::GFP11 + pan-somatically expressed GFP1-10. (B, C) Example images (B) and quantification (C) of control vs. auxin-treated animals carrying the pan-neuronal TIR1, the endogenously-tagged HLH-30::GFP11::AID allele, and a PVD>GFP1-10 transgene, which show that the pan-neuronal TIR1 strain depletes HLH-30 from the PVD neuron. Scale bar = 50 μm (A) or 10 μm (B). The data underlying the graphs shown in the figure can be found in S1 Data. (PDF) [file pbio.3002957.s003.pdf]

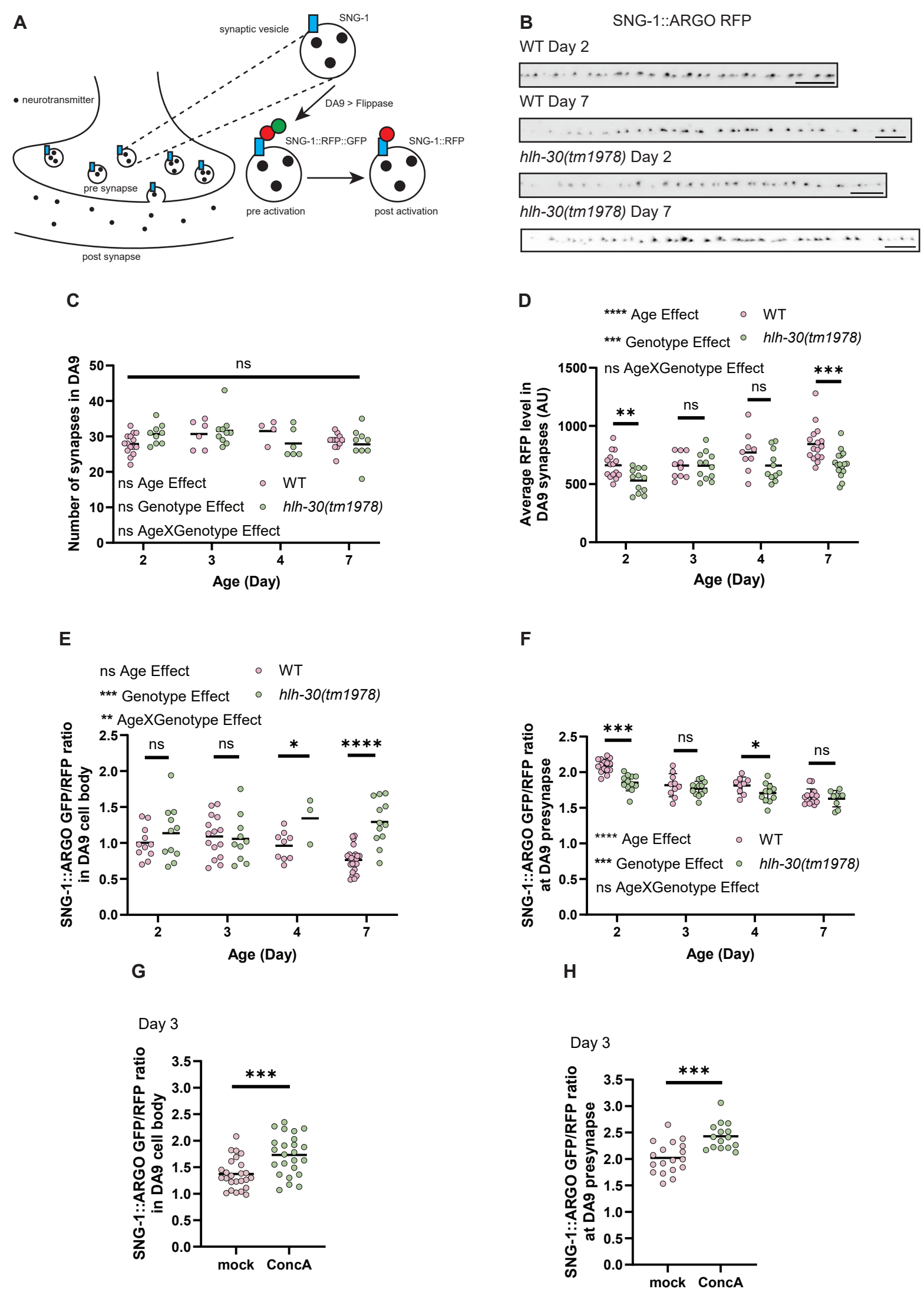

Fig S4

Supplement: S4 Fig — (A) Schematic representation of the ARGO method for quantifying SNG-1 turnover using ratiometric fluorescence imaging. In DA9 neurons, SNG-1 are tagged with both GFP and RFP before activation. After activation, newly synthesized SNG-1 proteins are tagged with only RFP. SNG-1 protein half-life can be measured by the decrease of relative GFP to RFP ratio over time. (B–D) DA9 presynapses shows no apparent difference in number or organization between WT and the hlh-30 mutant (C), but the SNG-1::ARGO RFP fluorescence intensity is slightly decreased in the hlh-30 mutant compared to WT (D). Scale bar = 10 μm. In (D), each data point shows the average of the maximum RFP intensity of all SNG-1::ARGO across all the presynapses in each worm. ***P < 0.001, **P < 0.01, ns: not significant, Two-way ANOVA with Tukey post-test. (E, F) Steady-state GFP/RFP ratio of SNG-1::ARGO in the cell body (E) and presynapses (F). The lower steady-state SNG-1::ARGO GFP/RFP ratio within vesicles in the cell body compared to at the synapses shows that the cell body vesicles are acidic lysosomal compartments. (E) The cell body SNG-1::ARGO GFP/RFP ratio is increased in the hlh-30 mutant compared to WT at adult Day 4 and 7. Each data point shows the GFP/RFP ratio of SNG-1::ARGO-labeled endosome in the cell body. ****P < 0.0001, *P < 0.05, ns: not significant, Two-way ANOVA with Tukey post-test. (F) Steady-state SNG-1::ARGO GFP/RFP ratio at the synapses is decreased in the hlh-30 mutant compared to WT at Day 2 and 4. Each data point is the average GFP/RFP from all presynapses within a single neuron, within a single animal. Note that the steady-state GFP/RFP ratio at the synapses is near two rather than one because GFP is brighter than RFP. ***P < 0.001, *P < 0.05, ns: not significant, linear mixed-effects model with Tukey post-test. Comparisons were performed on log-transformed data to meet model assumptions. (G-H) Day 3 worms injected with concanamycin A and imaged 3 hours post-injection showed an in [file pbio.3002957.s004.pdf]

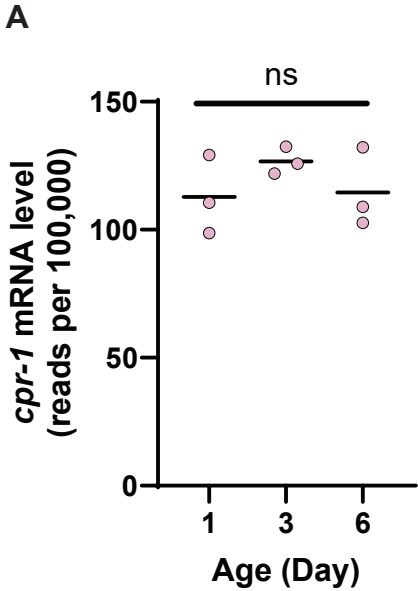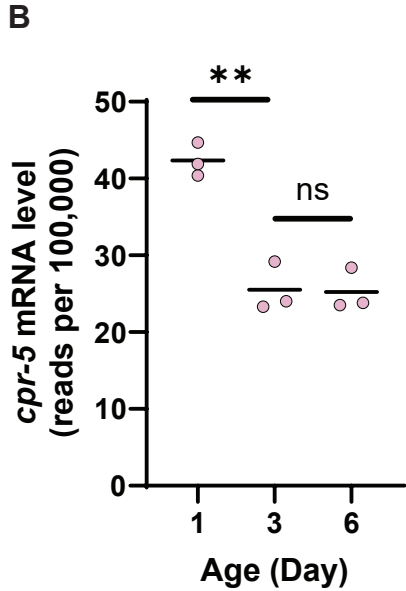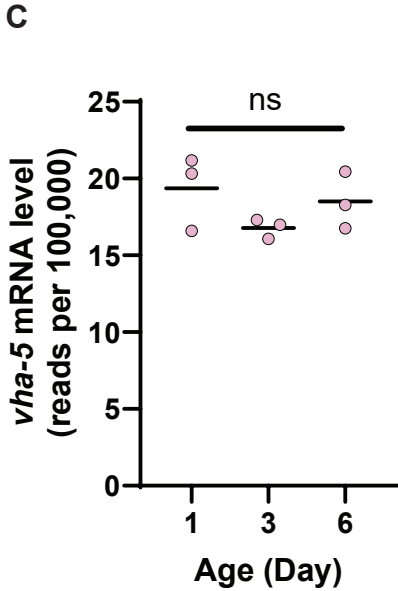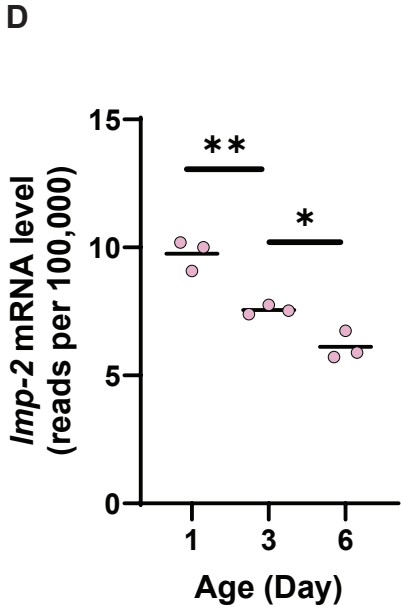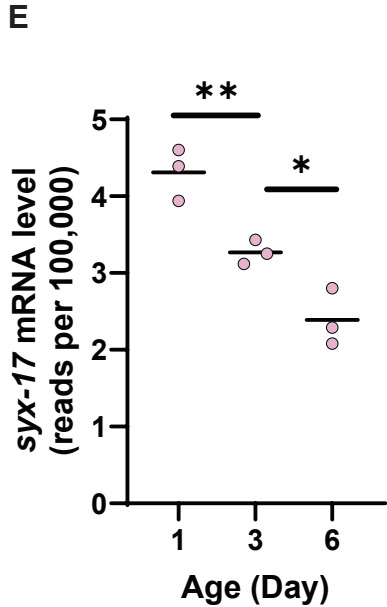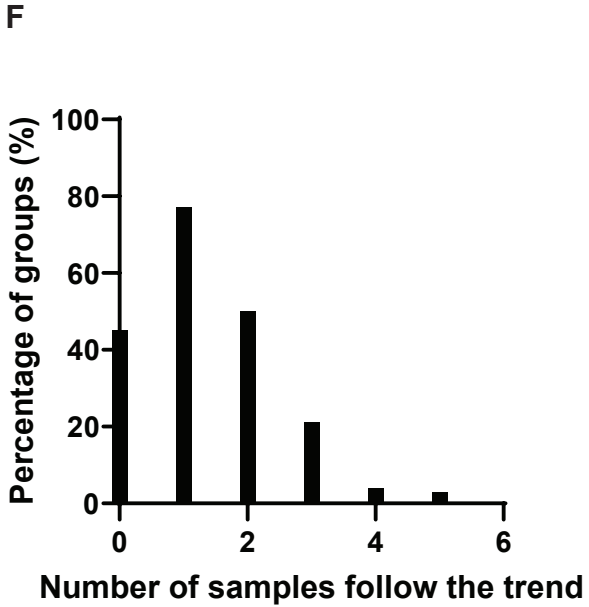

Fig S6

Supplement: S6 Fig — (A–E) In addition to rab-7 (Fig 3G), three out of five more HLH-30-regulated genes show reduced mRNA level along with aging. **P < 0.01, *P < 0.05, ns: not significant, One-way ANOVA with Tukey post-test [60]. (F) Bootstrapping analysis showing numbers of genes in randomly selected groups of 6 genes that have reduced mRNA level with aging. The data underlying the graphs shown in the figure can be found in S1 Data. (PDF) [file pbio.3002957.s006.pdf]

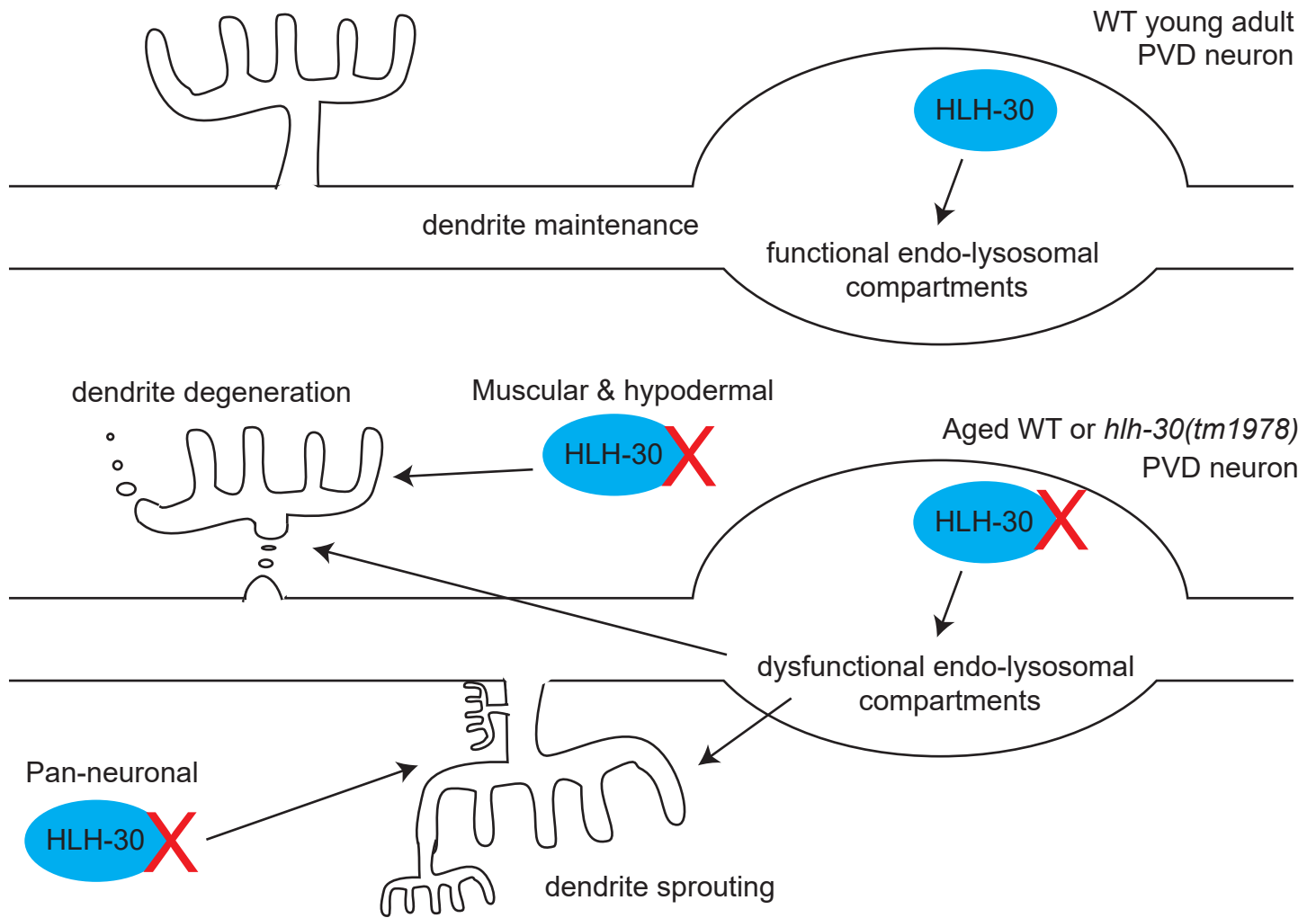

Fig S7

Supplement: S7 Fig — In young adult animals, basal HLH-30 level increases to expand endo-lysosomal degradative capacity, maintaining dendrite integrity. In the hlh-30 mutant or aged adult worms, reduced HLH-30 activity leads to inadequate lysosomal functions, causing aberrant dendrite morphology phenotypes in PVD neurons. Neuronal, muscular and hypodermal HLH-30 activity is required to protect the PVD dendrite from degeneration, whereas only neuronal HLH-30 helps prevent dendrite sprouting defect. (PDF) [file pbio.3002957.s007.pdf]
