# Supplementary material for: Expansion of lysosomal capacity in early adult neurons driven by TFEB/HLH-30 protects dendrite maintenance during aging in Caenorhabditis elegans
Source: PLoS Biol. 2025 Sep 30;23(9):e3002957. doi: 10.1371/journal.pbio.3002957 (PMC12510649; doi:10.1371/journal.pbio.3002957)
Supplement: S1 Table — (DOCX) [file pbio.3002957.s008.docx]

**Supplement Table 1**

The following strains were generated in the Richardson lab:

| Strain name | Genotype | Figures |
| --- | --- | --- |
| PBT26 | *wyIs592 (Pser2prom3::myr-gfp,Podr-1::rfp) III; hlh-30(tm1978) IV; wyIs853 (Pser2prom3::mCherry::rab-7,Podr-1::gfp) V* | Figs 2A-C, 4A-G and 4J-K |
| PBT60 | *him-5(e1490) V; carEx6 (Pser2prom3::nuc-1::rfp, Pser2prom3::nuc-1::gfp, Pdes-2::bfp, Podr-1::gfp)* | Figs 1A-D, 2D-H, S1A and S2D-F |
| PBT67 | *carIs1 (Pglr-4::flp, Podr-1::rfp) II; sng-1 (syb3140car2[sng-1::FLPon(FRT)::rfp::loxP::gfp::loxP]), heSi160 (Phsp-16.48::Cre) X* | Figs 2I, S1C, S4 and S5A-F |
| PBT115 | *hlh-30(tm1978) IV; carEx6 (Pser2prom3::nuc-1::rfp, Pser2prom3::nuc-1::gfp, Pdes-2::bfp, Podr-1::gfp)* | Figs 1B, 2D-H and S1A |
| PBT118 | *snt-1 (ox698[gfp::FLPon(FRT)::snt-1]) II; wyIs836 (Pnhr-81::flp); carEx4 (Pser2prom3::nuc-1::rfp, Pdes-2::bfp, Podr-1::gfp) line 1* | Figs 1E-H, S1B and S2A |
| PBT127 | *rab-7(wy1390[GFP::FLPon(FRT)::rab-7]) II; hlh-30(tm1978) IV; wyIs910 (Pser2prom3::flp; Punc-122::bfp) X* | S2C Fig |
| PBT131 | *wyIs592 (Pser2prom3::myr-gfp, Podr-1::rfp), cup-5(ar465) III; wyIs853 (Pser2prom3::mCherry::rab-7, Podr-1::gfp) V* | Fig 4H-I |
| PBT132 | *wyIs592 (Pser2prom3::myr-gfp, Podr-1::rfp) III; hlh-30(tm1978) IV; wyIs853 (Pser2prom3::mCherry::rab-7, Podr-1::gfp) V; carEx10 (Pser2prom3::hlh-30::gfp, Punc-122::rfp)* | Figs 2A, 2C and 4F-G |
| PBT146 | *wrdSi23 (Peft-3::TIR1::F2A::mTagBFP2::AID*::nls::tbb-2 3'UTR) I; wyIs592 (Pser2prom3::myr-gfp, Podr-1::rfp) III; hlh-30(syb9347) IV; wyIs853 (Pser2prom3::mCherry::rab-7, Podr-1::gfp) V* | Figs 4J-K and S1F |
| PBT150 | *wyIs853 (Pser2prom3::mCherry::rab-7, Podr-1::gfp) V; carEx10 (Pser2prom3::hlh-30::gfp, Punc-122::rfp)* | Fig 2C |
| PBT159 | *wyIs592 (Pser2prom3::myr-gfp, Podr-1::rfp) III; wyIs853 (Pser2prom3::mCherry::rab-7, Podr-1::gfp) V; carEx10 (Pser2prom3::hlh-30::gfp, Punc-122::rfp)* | Fig 4L-M |
| PBT161 | *rab-7(wy1390[GFP::FLPon(FRT)::rab-7]) II; wyIs910 (Pser2prom3::flp; unc-122::bfp) X* | S2C Fig |
| PBT166 | *muIs253 (Peft-3::sfGFP1-10::unc-54 3'UTR, Cbr-unc-119(+)), rab-7 (car10[gfp-11::nls::PEST::sl2::rab-7]) II; carEx4(Pser2prom3::nuc-1::rfp, Pdes-2::bfp, Podr-1::gfp) line 1* | Figs 3E-F and S1E |
| PBT170 | *hlh-30(syb9347) IV; wyIs853 (Pser2prom3::mCherry::rab-7, Podr-1::gfp) V; juEx4586(Prgef-1::gfp1-10, Pttx-3::rfp)* | Figs 3A-B and S1D |
| PBT171 | *wyIs592 (Pser2prom3::myr-gfp, Podr-1::rfp) III; hlh-30(tm1978) IV; wyIs853 (Pser2prom3::mCherry::rab-7, Podr-1::gfp) V; carEx12 (Punc-119::hlh-30::gfp)* | Fig 4F-G |
| PBT172 | *snt-1 (ox698[gfp::FLPon(FRT)::snt-1]) II; wyIs836 (Pnhr-81::flp); hlh-30(tm1978) IV; carEx4 (Pser2prom3::nuc-1::rfp, Pdes-2::bfp, Podr-1::gfp) line 1* | Figs 1F, 1H and S1B |
| PBT173 | *reSi3 (Punc-54::TIR1::F2A::mTagBFP2::AID*::nls::tbb-2 3'UTR) I; wyIs592 (Pser2prom3::myr-gfp, Podr-1::rfp) III; hlh-30(syb9347) IV; wyIs853 (Pser2prom3::mCherry::rab-7, Podr-1::gfp) V* | Figs 4J-K and S1F |
| PBT174 | *reSi5 (Pges-1::TIR1::F2A::mTagBFP2::AID*::nls::tbb-2 3'UTR) I; wyIs592 (Pser2prom3::myr-gfp, Podr-1::rfp) III; hlh-30(syb9347) IV; wyIs853 (Pser2prom3::mCherry::rab-7, Podr-1::gfp) V* | Figs 4J-K and S1F |
| PBT183 | *muIs253 (Peft-3::sfGFP1-10::unc-54 3'UTR, Cbr-unc-119(+)) I, rab-7(car10) II; hlh-30(tm1978) IV; carEx4(Pser2prom3::nuc-1::rfp, Pdes-2::bfp, Podr-1::gfp) line 1* | Figs 3F and S1E |
| PBT184 | *reSi2 (Pcol-10::TIR1::F2A::mTagBFP2::AID*::nls::tbb-2 3'UTR) II; wyIs592 (Pser2prom3::myr-gfp, Podr-1::rfp) III; hlh-30(syb9347) IV; wyIs853 (Pser2prom3::mCherry::rab-7, Podr-1::gfp) V* | Figs 4J-K and S1F |
| PBT185 | *reSi7(Prgef-1::TIR1::F2A::mTagBFP2::AID*::NLS::tbb-2 3'UTR) I; wyIs592 (Pser2prom3::myr-gfp, Podr-1::rfp) III; hlh-30(syb9347) IV; wyIs853 (Pser2prom3::mCherry::rab-7, Podr-1::gfp) V* | Figs 4J-K and S1F |
| PBT186 | *carIs1 (Pglr-4::flp, Podr-1::rfp) II; hlh-30(tm1978) IV; sng-1 (syb3140car2[sng-1::FLPon(FRT)::rfp::loxP::gfp::loxP]), heSi160 (Phsp-16.48::Cre) X* | Figs 2I, S1C, S4B-F, S5A, S5C-E and S5G |
| PBT207 | *reSi7 (Prgef-1::TIR1::F2A::mTagBFP2::AID*::nls::tbb-2 3'UTR) I; hlh-30 (syb9347) IV; carEx6 (Pser2prom3::NUC-1::RFP, Pser2prom3::NUC-1::GFP, Pdes-2::BFP, Podr-1::GFP)* | Figs 2H, S1A and S1F |
| PBT213 | *wyIs592 (Pser2prom3::myr-gfp, Podr-1::rfp) III; hlh-30(tm1978) IV; wyIs853 (Pser2prom3::mCherry::rab-7 ,Podr-1::gfp) V; carEx13(Peft-3::hlh-30(isoform c)::GFP)* | Fig 4F-G |
| PBT214 | *reSi7(rgef-1p::TIR1::F2A::mTagBFP2::AID*::nls::tbb-2 3'UTR) I; shyIs? (Pser-2prom3::mCD8::RFP, Pser-2prom3::spGFP1-10, Podr-1::RFP); hlh-30 (syb9347) IV* | S1F and S3B-C Figs |
| PBT215 | *wrdSi23 (Peft-3::TIR1::F2A::mTagBFP2::AID*::nls::tbb-2 3'UTR) I; muIs253 (Peft-3::sfGFP1-10::unc-54 3'UTR, Cbr-unc-119(+)) II; hlh-30 (syb9347) IV* | S1F and S3A Figs |
| PBT216 | *reSi2 (Pcol-10::TIR1::F2A::mTagBFP2::AID*::nls::tbb-2 3'UTR), muIs253 (Peft-3::sfGFP1-10::unc-54 3'UTR, Cbr-unc-119(+)) II; hlh-30 (syb9347) IV* | S1F and S3A Figs |
| PBT217 | *reSi3 (unc-54p::TIR1::F2A::mTagBFP2::AID*::nls::tbb-2 3'UTR) I; muIs253 (Peft-3::sfGFP1-10::unc-54 3'UTR, Cbr-unc-119(+)) II; hlh-30 (syb9347) IV* | S1F and S3A Figs |
| PBT218 | *reSi5 (Pges-1::TIR1::F2A::mTagBFP2::AID*::nls::tbb-2 3'UTR) I; muIs253 (Peft-3::sfGFP1-10::unc-54 3'UTR, Cbr-unc-119(+)) II; hlh-30 (syb9347) IV* | S1F and S3A Figs |
| PBT219 | *reSi7 (Prgef-1::TIR1::F2A::mTagBFP2::AID*::nls::tbb-2 3'UTR) I; muIs253 (Peft-3::sfGFP1-10::unc-54 3'UTR, Cbr-unc-119(+)) II; hlh-30 (syb9347) IV* | S1F and S3A Figs |

The following strains were provided by Shen lab, Stanford University:

| Strain name | Genotype | Figures |
| --- | --- | --- |
| TV25223 | *wyIs592 (Pser2prom3::myr-gfp, Podr-1::rfp) III; wyIs853 (Pser2prom3::mCherry::rab-7, Podr-1::gfp) V* | Figs 2A-C and 4 |

The following strains were provided by the CGC:

| Strain name | Genotype | Figures |
| --- | --- | --- |
| N2 | WT | S2B Fig |
| JIN1375 | *hlh-30(tm1978) IV* | S2B Fig |
