# Supplementary material for: Expansion of lysosomal capacity in early adult neurons driven by TFEB/HLH-30 protects dendrite maintenance during aging in Caenorhabditis elegans
Source: PLoS Biol. 2025 Sep 30;23(9):e3002957. doi: 10.1371/journal.pbio.3002957 (PMC12510649; doi:10.1371/journal.pbio.3002957)
Supplement: S2 Table — (DOCX) [file pbio.3002957.s009.docx]

**Supplement Table 2**

The following primers were used for cloning:

| Gene | Primer | Sequence |
| --- | --- | --- |
| *nuc-1* | pRZ1insF | AAggcgcgcc caggtgagggagggtaagag |
|  | pRZ1insR | AAGGTACCGGtgcacaattattttgggttgc |
| *rab-7* | pRZ2insF | AAAAAGGATCC ATGTCGGGAACCAGAAAGAA |
|  | pRZ2insR | AAAAGGTACC ggagacgagggaagaggaaa |
| *hlh-30* | pRZ13insF | AAAAGGTACC ATGATCCGCCAACTAAA |
|  | pRZ13insR | AAAAGGTACC gcCGAAAAGTCCATGTGATAA |

The following primers were used for genotyping:

| Allele | Primer | Sequence |
| --- | --- | --- |
| *rab-7(wy1390)* | rab-7flp-onF | GGTTCCTGACATGCTTCCAC |
|  | rab-7flp-onR | CACATGGTCCTCCTCGAGTT |
| *hlh-30(tm1978)* | pRZ4seqF2 | ccggatgaggactgaaacat |
|  | pRZ4seqR | attttgcgccaaaaattgtc |
| *hlh-30(syb9347)* | pRZ1criF1 | AGACCCGATCAACAC |
|  | pRZ1criR3 | ATGTATGTACAGGAACG |
