# Supplementary material for: Expansion of lysosomal capacity in early adult neurons driven by TFEB/HLH-30 protects dendrite maintenance during aging in Caenorhabditis elegans
Source: PLoS Biol. 2025 Sep 30;23(9):e3002957. doi: 10.1371/journal.pbio.3002957 (PMC12510649; doi:10.1371/journal.pbio.3002957)
Supplement: S5 Fig — (A) Representative image of SNG-1::ARGO GFP, RFP channel in the DA9 cell body shows increased GFP fluorescence in aged hlh-30 mutant. Scale bar = 10 μm. (B) Example images of presynaptic SNG-1::ARGO GFP, RFP, and the GFP/RFP ratio imaged at the indicated ages. For the Day 7 post-activation animal, activation occurred on Day 2 of adulthood. In the GFP/RFP ratio images, the color scale ranges from dark blue (low GFP/RFP) to pale yellow (high GFP/RFP). Scale bar = 10 μm. (C) Line scan images of the RFP channel in DA9 neuron synapses labeled by SNG-1::ARGO shows no apparent difference in synapse organization in the hlh-30(tm1978) mutant compared to WT. In the GFP/RFP ratio images, the color scale ranges from dark blue (low GFP/RFP) to pale yellow (high GFP/RFP). Scale bar = 10 μm. (D, E) The hlh-30 mutant shows decreased SNG-1::ARGO puncta size (D) and fewer puncta (E) in the neuron cell body by Day 7 compared to WT. **P < 0.01, *P < 0.05, two-tailed Student t test. (F, G) Quantification of SNG-1::ARGO GFP/RFP ratio at the synapse after the heat shock “pulse” in WT (F) and the hlh-30(tm1978) mutant (G). Each data point shows the average GFP/RFP ratio from all presynapses within a single neuron. These data were used to generate the decay curves for Fig 2I. The data underlying the graphs shown in the figure can be found in S1 Data. (PDF) [file pbio.3002957.s005.pdf]

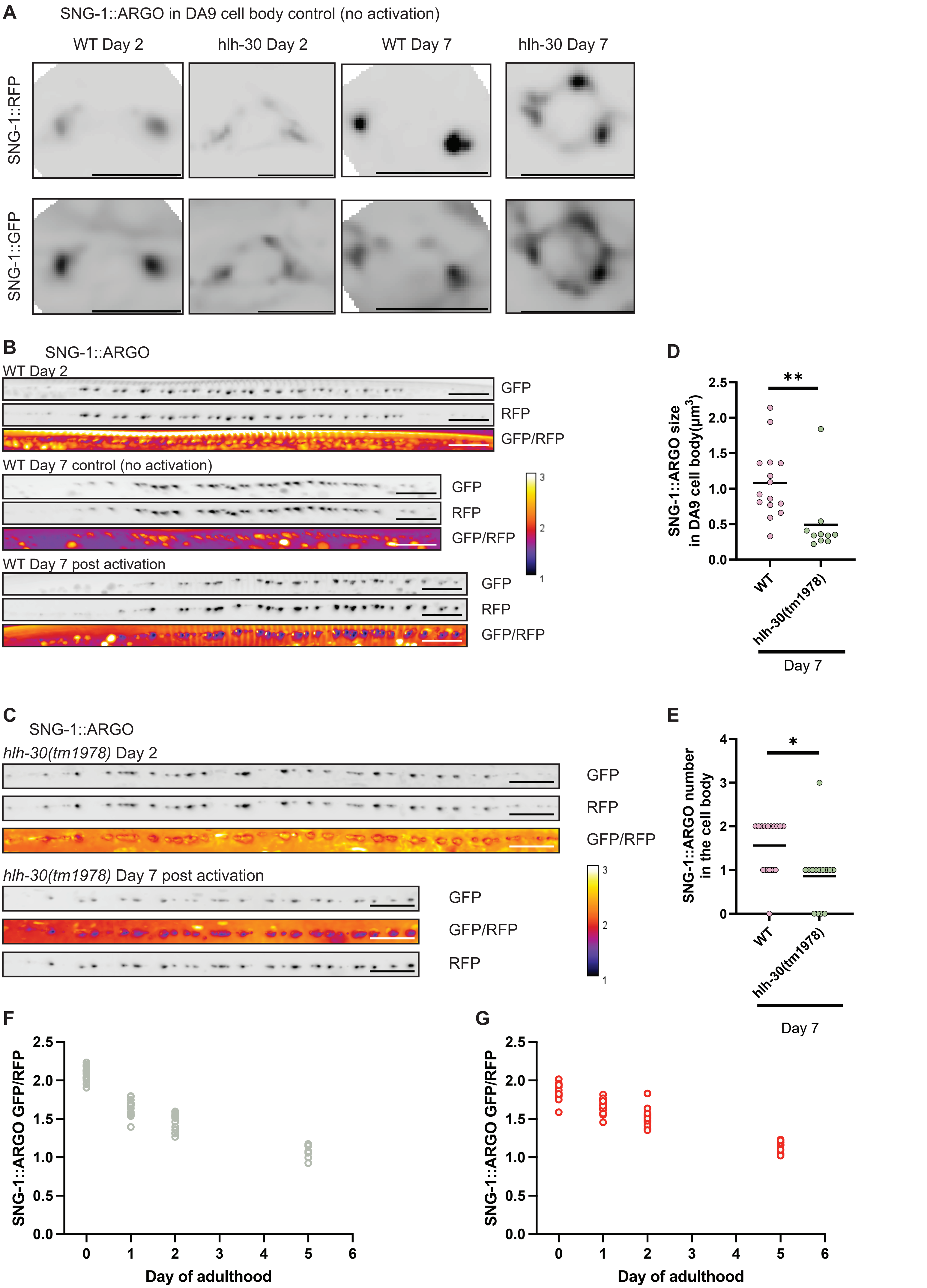
A
SNG-1::ARGO in DA9 cell body control (no activation)
WT Day 2
hlh-30 Day 2
WT Day 7
hlh-30 Day 7
SNG-1::RFP
SNG-1::GFP
B
SNG-1::ARGO
WT Day 2
GFP
RFP
GFP/RFP
WT Day 7 control (no activation)
WT Day 7 post activation
C
SNG-1::ARGO
hlh-30(tm1978) Day 2
hlh-30(tm1978) Day 7 post activation
D
SNG-1::ARGO size in DA9 cell body(μm³)
**
WT
hlh-30(tm1978)
Day 7
E
SNG-1::ARGO number in the cell body
*
F
SNG-1::ARGO GFP/RFP
Day of adulthood
G
SNG-1::ARGO GFP/RFP
Day of adulthood

Fig S5
