# Supplementary material for: Expansion of lysosomal capacity in early adult neurons driven by TFEB/HLH-30 protects dendrite maintenance during aging in Caenorhabditis elegans
Source: PLoS Biol. 2025 Sep 30;23(9):e3002957. doi: 10.1371/journal.pbio.3002957 (PMC12510649; doi:10.1371/journal.pbio.3002957)
Supplement: Attachment — Submitted filename: Zhong et al Response to Reviewers_fixed.docx [file pbio.3002957.s011.docx]

Rev. 1:

In this manuscript, the authors report age-related lysosomal dysfunction in C. elegans neurons, and discovered that the worm TFEB homolog, HLH-30, as a contributor to this change. They first generated fluorescent transgenic reporters to measure lysosomal pH-dependent quenching and cargo degradation in PVD neurons. Using these reporters, the authors found that lysosomal degradation decreases while lysosomal size increases with age. Next, they examined the involvement of HLH-30 and found that its loss accelerates lysosomal dysfunction during aging. Furthermore, the authors showed that HLH-30 nuclear localization is not affected by aging, but its transcriptional level decreases together with the transcriptional decrease of lysosomal genes. Lastly, they detected interesting age-related morphological changes in PVD dendrites, including increased degeneration and sprouting, and identified the potential role of HLH-30 and lysosomal dysfunction in these processes. Overall, the

manuscript is well-written and presents interesting findings. However, the rigor of the results needs to be be strengthened, and several major concerns require further experimental evidence.

Major points:

1. The choice of worm ages for different analyses varies considerably across the manuscript, which makes it difficult to fully assess the temporal patterns of age-related changes in WT and mutant worms.

a. Morrimoto's prior work showed that neuronal dysfunction occurs as early as day 2 of adulthood (Ben-Zvi et al 2009). The authors should assess lysosomal activity starting at day 1 and include additional time points between day 6 and day 9 (in Figs. 1B, 1C, 1E, 2E). In addition, the authors should discuss whether the observed lysosomal dysfunction is a consequence of neuronal dysfunction, or a driver of it.

For Fig 1B, we added time points at Day 1, Day 7 and Day 8. We split the data and analysis of lysosome acidity in WT Ctrl vs ConcA vs hlh-30 and WT across aging into two figure panels, Fig 1B and 1C. For 1C, in response to this reviewer’s comment #4, we took images using super resolution microscopy, and we replaced the data with the results we obtained with higher resolution and added timepoints at Day 1, 6, and 11 (see also response to Major points #3 and #4). We moved the lysosome volume analysis to Figs 2G-H. We moved it because we think Fig 1 is mainly testing lysosome function, and according to our new results obtained with super resolution imaging, lysosome volume is not simply correlated with lysosomal dysfunction. For 1E, we added analyses at Day 1 and Day 6. For original 2E, we added a time point at Day 1, and combined it into Fig 1B.

The model we put forward is that lysosomal dysfunction is a driver of neuronal dysfunction. Some key evidence supporting that notion is that impairing lysosomal function with either chronic concanamycin A treatment or the *cup-5* mutant accelerates the age-associated dendrite morphology defects. If lysosomal dysfunction increased with adult age but was not a driver of the dendrite morphology defects, we would likely observe no change in the timing of when these defects arise when we inhibit lysosomal function. (lines 506 - 518, 572-574). Our model also proposes that declining HLH-30 activity is one of the drivers for lysosome dysfunction, and that there is one or more HLH-30-independent mechanism that leads to declining lysosome function with age. We added this consideration of a parallel pathway in lines 438 - 444. We adjusted the wording of our reference to the Ben-Zvi et al., 2009 in the discussion to clarify that it seems as though the lysosomal dysfunction we observe and the declining proteostasis they observe (including of cargoes for proteasomal degradation) happen in parallel (lines 598 - 600). The original wording suggested that declining lysosomal function likely contributes to both impaired autophagic flux and declining proteostasis, and we still think that is the case (lines 598 – 600).

There seems to be a circular relationship between neuronal aging and lysosomal dysfunction. We added a paragraph to the discussion pointing out that aging also seems to cause neuronal lysosomal dysfunction (lines 601-605).

b. The rationale for choosing day 2 and day 7 in Figs. 2H, 2I is unclear. Data from additional time points should be provided.

We added time points at Day 3 and Day 4 for Figs. 2H and 2I, and those panels are now Fig 3I and Fig 3J. This is because we split old Fig 2 into two figures, with new Fig 2 containing all PVD neuron results, and new Fig 3 containing all DA9 neuron results. For these two figure panels, we were using the same strain (PBT67) and assessing the same neuron (DA9) as Figs, 2J, 2K, and 2L (now Figs 3K, 3L, 3M), which follows the method from another paper from our lab (Shiliaev et al., 2024 doi: <https://doi.org/10.1101/2024.11.26.625560>). We decided to use the same timepoint from that paper for all the DA9 neuron-related experiments to assess and ensure reproducibility.

c. Given that the lysosomal size and cargo accumulation difference become evident only between day 3 and day 9, the authors should include day 9 comparison in Figs. 2F, 2G beyond day 6. It is particularly important as no significant difference is observed in day 6 but trends appear.

We replaced the measurement of lysosomal size from Fig 2F using a higher resolution to increase the accuracy (see also response to major comment #4). We added a timepoint for comparing WT vs hlh-30 at Day 6 (Figs 2G-H). We also changed the statistical analysis of individual lysosome size and SNT-1::GFP to a linear mixed-effects model. This is because for both results, we have multiple measurements from each animal, so the animal is the random effect in the linear mixed-effects model. Using these more accurate data and statistical analysis, we found that there is a significant difference between WT and the *hlh-30* mutant at Day 3 and Day 6 (Fig 2G). However, whereas our originally submitted manuscript reported no statistically significant difference in lysosomal compartment size with a trend for increased size in the *hlh-30* mutant, we now show that lysosomes in the *hlh-30* mutant are, in fact, smaller compared with WT at adult Day 3 and 6. We also observed the same trend of reduced lysosome size under ConcA treatment, suggesting the increasing lysosome size is not simply anti-correlated with lysosome function. We also analyzed the total lysosomal compartment volume within each neuron cell body. In WT, the total lysosomal compartment volume significantly increases from Day 1 to Day 3 of adulthood, whereas in the *hlh-30* mutant, the total lysosomal compartment volume does not change between Day 1 and Day 3. This suggests that *hlh-30* is required for lysosomal capacity expansion in early adulthood. (See line 251-271, Figs 2F-H).

We included Day 6 data for Fig 2G, and combined the data into Fig 1E. There is no significant difference between any pairwise comparison between WT and the *hlh-30* mutant at all ages when simply comparing the SNT-1::GFP intensities within lysosomal compartments. After plotting all SNT-1::GFP fluorescence intensity in WT, and in response to this reviewer’s major point #3, we noticed that SNT-1::GFP intensities from WT lysosomal compartments follows a bimodal distribution, suggesting that there are two different groups of lysosomes (Fig 1F). We used this bimodal distribution to set a threshold in the thought to assess the proportion of lysosomal compartments with atypically high levels of SNT-1::GFP (Figs 1F-G). This analysis showed that both the *hlh-30* mutant and chronic concanamycin treatment increased the proportion of atypical lysosomal compartments at Day 3 (Fig 1G). By Day 6, the WT animals also show a similar increase in the proportion of atypical lysosomal compartments, at which point the WT, concanamycin A-treatment, and *hlh-30* mutants become indistinguishable. (See lines 178-191, 295-301, Fig 1E-G). After doing a power analysis for these dataset, we found that we need at least 50 worms for each time point, and it was not feasible to analyze 50 *hlh-30* mutants for a Day 9 timepoint with our manpower and other resources.

d. It is shown that concanamycin A induces lysosomal dysfunction at day 6. Does this effect only occur at this later time point? Given that this is a crucial validation for the reporters, the authors should measure the effect of concanamycin A at other ages.

We measured lysosome acidity under concanamycin A treatment at Day 3 and added the result to Fig. 1B. There’s no significant difference between Day 3 Ctrl vs ConcA. This does not rule out the possibility that concanamycin A begins to induce lysosomal dysfunction by Day 3 but this particular experiment is not sensitive enough to detect it.

2. The authors should avoid reusing WT data for comparison. For comparing WT and the hlh-30(o) mutant, data from experiments conducted together should be shown, rather than reusing WT data from Fig 1.

We repeated the experiments shown in the original Fig.1B and replaced it with some new data such that WT data is not repeated, the new results are shown in Fig 1C. For the original Fig 1C, we replaced the measurement of lysosomal size using a higher resolution to increase the accuracy (see also response to major comment #4), and combine the WT, *hlh-30* mutant and concA treatment data together to avoid repeating WT data. We repeated some new experiment for SNT-1 degradative reporter, and combine WT, *hlh-30* mutant and concA treatment data together into Fig 1E to reduce the reuse of WT data.

3. In images shown in Fig. 1D, there are both normal and abnormal lysosomes in the same cell body. In Fig. 1E, day 9 samples seem to contain two populations. So, it might be informative to calculate the percentage of abnormal lysosomes per cell body at different ages.

As described above in response to point 1c, we plotted the WT data of Fig 1E and found there are indeed two groups of lysosomes in the same cell body (Fig 1F). We quantified the fraction of lysosomes with high SNT-1::GFP within each worm and quantified the percentage of atypical lysosomes among all lysosomes (Fig 1G).

4. Lysosomes larger than 10um appear unusually large and likely represent clusters as mentioned by the authors. This may indicate an age-related increase in lysosomal fusion/clustering. The authors should consider using higher-resolution microscopy to distinguish individual lysosomes more clearly.

We are especially grateful for this suggestion. We took images of lysosomes using super resolution fluorescence confocal microscopy, and we were able to distinguish individual lysosomes within clusters, which we believe substantially improved the accuracy of our quantifications (Fig S2A). We replaced the lysosome volume counts with our new quantifications (Figs 2G-H). Most of the volumes of individual lysosomes are smaller than 10 um with this improved imaging technique.

5. When showing PVD rescuing effects, the authors should include a critical control without the hlh-30 mutant in the background. In addition, the authors should provide the statistical result for the comparison between day 0 and day 3 in the hlh-30(0) mutant. This comparison is important to determine whether there is still an age-related increase even without hlh-30. Furthermore, the authors should provide PVD rescuing results for SNT-1::GFP and NUC-1::1GFP/RFP ratio. These results are essential to determine whether PVD hlh-30 contributes to the observed lysosomal dysfunction.

We added a critical control with HLH-30 OE in the WT background, and there is no difference in late endosome numbers between WT and WT+HLH-30 OE at Day 3 (Fig 2C).

We added the statistical comparison for late endosome numbers at Day 0 vs. Day 3 in the *hlh-30* mutant. There is a significant difference in the *hlh-30* mutant between Day 0 and Day 3, suggesting that the late endosome number increases somewhat in early adulthood even without HLH-30 function (Fig. 2B).

We used PVD>GFP::HLH-30 for the PVD-specific rescue experiment shown in Fig. 2B, and since we measured GFP intensity in both lysosome acidity and lysosome degradative capacity reporters, we are not able to use the GFP::HLH-30 allele for rescuing experiment. Instead, we measured lysosome acidity in WT versus the *hlh-30* mutant versus HLH-30 pan-neuronal depletion using auxin-inducible degradation (Fig 2I). The result suggests that HLH-30 functions at least in part from the neurons to promote proper lysosomal compartment acidity (Fig 2I).

6. In Fig. 3F, the authors should conduct age-related comparisons in the hlh-30(o) mutant, especially between day 3 and day 6. It seems that the age-related decrease also occurred without hlh-30. If so, it would suggest other factors contribute to the age-related decrease, while hlh-30 only mediates general decreases.

We added comparisons between Day 0, Day 3, and Day 6 in the *hlh-30* mutant. There is indeed a significant difference in *rab-7* transcription level between Day 3 and Day 6 in the hlh-30 mutant (Fig 4F). In WT, the fluorescence intensity of our *rab-7* transcriptional reporter drops by 40 ± 5%, and in the *hlh-30* mutant, it declines by 26 ± 4%. These results suggest that there *rab-7* transcription declines between Day 3 and 6 through both HLH-30-dependent and -independent mechanisms (Fig. 4F). We describe these results in lines 437 – 444.

7. The transcriptional decrease of hlh-30 begins at day 3 (Fig. 3H), which is consistent with decreases in lmp-2, cpr-5, and syx -17 (Fig. S4B, D, E). However, the decrease of rab-7 transcription only started at day 6 (Fig. 3G). Together with point #6, it is likely that other factors may contribute to the age-related transcriptional decrease of rab-7.

We included discussion about other factors might also contribute to the age-related decrease of rab-7 transcription (lines 438 – 444).

8. It is clear from the data shown in Figure 4, dendrite degeneration and sprouting are regulated by different mechanisms: 1. PVD specific rescue of hlh-30 only rescued degeneration but not sprouting. 2. Neuronal, hypodermal, and muscular loss of HLH-30 all resulted in dendrite degeneration, while only neuronal loss of HLH-30 caused dendrite sprouting. 3. PVD specific overexpression of hlh-30 attenuated age-related dendrite sprouting but not its degeneration. Therefore, the authors should separate these two processes and refine the model to summarize their findings.

We changed the discussion by separating the degeneration and sprouting phenotype (lines 575-586, see also lines 635 - 661). We also separated the two processes in the model (Fig. 6).

9. Without demonstrating that PVD-specific rescuing of HLH-30 attenuates the age-related lysosomal dysfunctions (see point #5), the proposed model cannot be fully supported. Especially, the dendrite degeneration does not appear directly linked to HLH-30 function in PVD, regardless of its role in regulating age-related lysosomal dysfunction.

Based on Figs 5F and 5J (original Figs 4D and 4H), both PVD-specific and pan-neuronal HLH-30 are able to partially rescue the degeneration phenotype in the *hlh-30* mutant, and pan-neuronal depletion HLH-30 phenocopies the *hlh-30* mutant. The PVD-specific rescue of the degeneration phenotype indicates that HLH-30 functions cell-intrinsically, in part, to prevent dendrite degeneration. Our tissue-specific depletion data also indicate that it has non-cell-autonomous roles (current Fig 5J).

Based on Figs 5G and 5K (original Figs 4E and 4I), pan-neuronal HLH-30 can partially rescue the sprouting phenotype in the hlh-30 mutant and pen-neuronal depletion HLH-30 causes dendrite sprouting, suggesting that neuronal HLH-30 (but doesn’t have to be HLH-30 in PVD) is required for preventing dendrite sprouting.

To test where HLH-30 functions to promote neuronal lysosomal acidity, we did a pan-neuronal HLH-30 depletion experiment (Fig 2I). The results suggest that loss of neuronal HLH-30 accelerates the age-related lysosome dysfunction (Fig 2I and point #5). It remains possible that HLH-30 from a neuron or neurons other than the PVD neuron to promote lysosomal compartment acidity within the PVD neuron. We include this caveat in the description of the experiment (lines 292 – 294).

Minor points:

1. "Consistent with the framework that endolysosomal protein degradation occurs in the neuron cell body, we observed endosomes with a lower SNG-1::ARGO GFP/RFP fluorescence intensity ratio in the cell body compared to the presynapses in WT animals." Please revise this sentence as the comparison was not conducted between cell body and presynapses, but between WT and hlh-30(0) mutant.

We didn’t show the comparison in the same figure, so we rephrased that sentence (lines 353 - 360).

2. "Furthermore, in contrast to WT animals, we detected no difference in rab-7 transcription level between Day 0, Day 3, and Day 6 adults in hlh-30(o) mutants (Fig 3F)." This result was not shown.

We added the comparison (Fig. 4F) and changed the description (lines 438 – 444)

3. There is no Figure S4G (line 368).

We fixed that.

Rev. 2:

In this manuscript by Zhong and Richardson, the authors investigate the role of HLH-30, the C. elegans ortholog of mammalian transcription factor EB (TFEB), in maintaining neuronal health through the regulation of lysosomal function. While the study presents intriguing findings, including age-related changes in lysosomal compartment size and the role of hlh-30 in maintaining neuronal morphology, the conclusions are weakened by several critical issues. These include incomplete validation of experimental tools, missing control experiments, and claims about HLH-30-regulated gene expression trends with age that conflict with publicly available single-cell RNA-seq data. Furthermore, the description of HLH-30 localization and function under non-stress conditions requires revision for accuracy. Addressing these points would greatly enhance the manuscript's impact and its contribution to understanding HLH-30's role in lysosomal function and neuronal health.

Major comments:

Lysosome function declines during aging in C. elegans

- The ConcA validation experiment is important and can be used to assess reduced pH with age. Assessing the NUC-1::GFP/RFP ratio +/- ConcA in young animals (day 3 or 6) should show a larger increase after ConcA treatment compared to day 9 or 11, where the authors argue lysosomal pH has already increased. Additionally, ConcA treatment should also be applied to other reporters, including the SNG-1::GFP reporter and the RAB-7::mCherry reporter.

We added ConcA treatment to the SNT-1::GFP reporter (Fig 1E, 1G).

We measured lysosome acidity under concanamycin A treatment at Day 3 and added the result to Fig. 1B. There’s no significant difference between Day 3 Ctrl vs ConcA. This does not rule out the possibility that concanamycin A begins to induce lysosomal dysfunction by Day 3, but this experiment is not sensitive enough to detect it. It is also possible that it takes time for the drug ConcA to go through cuticles, get into PVD neurons and affect lysosome acidity in PVD neurons, so the pH level remains the same at Day 3.

- The authors should also assess lysosome number (as in Fig 2D) +/- ConcA treatment with age

We added ConcA treatment for lysosome numbers and size (current Figs 2F-H). Our data indicate that ConcA has a similar impact on these parameters as the *hlh-30* mutant, though the magnitude and timing are not identical.

- The authors should also include the RAB-7::mCherry reporter +/- ConcA treatment with age as shown in Fig. 2A-B.

We added ConcA treatment for RAB-7::mCherry reporter (Fig 2B). We found no statistically significant difference between Ctrl and ConcA in mCherry::RAB-7-labeled endosome numbers at Day 3, though there is a trend for a slight decrease. One possible reason that we do not detect a difference is that, as we mentioned above, it may take time for ConcA treatment to get through the cuticle and affect lysosome acidity in PVD neurons, so we might need to check a later timepoint to see the lysosome number change. Of note, our animals treated with ConcA show no detectable change in behavior as we do these experiments. If the ConcA was having a strong impact on the neurons, we would expect it to impede the loading of neurotransmitter into synaptic vesicles, thereby causing aberrant, or uncoordinated movement. This is evidence that the amount of ConcA that the neurons experience is indeed quite low. We added this note about animal behavior to the methods section to more clearly explain the experimental conditions (lines 767-771).

- Does PVD cell size change with age? Could age-related cell size changes correlate with the observed increase in lysosomal volume? PVD cell size should be measured using existing BFP images.

We measured PVD cell body size with the existing BFP images. Results show that PVD cell body size increases mainly between Day 3 to Day 6, but slightly decreases by Day 9. As both the total and individual lysosomal compartment size increases between Day 1 to 3 (Figs 2G-H), based on our improved quantification with super resolution microscopy, there is not a strong correlation between the age associated changes in lysosomal compartment size and neuron cell body size. We added this analysis as S2E Fig.

- Reassess the exposure levels for NUC-1::RFP in Fig. 1A and D and RAB-7::mCherry in Fig. 2A to ensure they are not overexposed. Include an outline of the cell body for all PVD neurons in Figs. 1 and 2 for clarity, as dendrites (?) are visible in Fig. 2, but not in Figure 1. Since this figure describes age-related changes in lysosomal compartments, include images of PVD neurons from aged animals (day 9 or 11) in Fig. 1A, similar to Fig. 1D.

HLH-30 is required for adult neuronal lysosomal capacity and function

No images were over-exposed during acquisition. Our confocal microscope is equipped with a CMOS camera, and our highest fluorescence intensity values for any of our fluorescence-tagged alleles tends to be on the order of 10,000 AU out of the possible 65,535.

We included an outline of the cell body for all PVD neurons in Figs 1 and 2 for clarity.

The dendrite is visible in Fig. 2A because there is some diffuse mCherry::RAB-7 in the cytoplasm. By contrast, NUC-1::RFP is trapped in the lumen of the lysosomal compartments.

There is no noticeable changes between young vs old animals in Fig 1A by eye, as the change in the GFP/RFP ratio is subtle. We included the aged images of PVD neurons (S2A Fig).

- Provide an explanation for the observed discrepancy in Fig. 2D, where lysosome numbers are reduced >3-fold in hlh-30 mutants, despite no significant difference in NUC-1::RFP volume on day 3 of adulthood.

Thank you for bringing this to our attention, this is an important discrepancy. The data for Fig 2D versus 2F were generated using two different alleles of NUC-1::RFP. We compared all of our alleles of NUC-1::RFP to each other and found that most alleles show far fewer NUC-1::RFP-labeled lysosomal compartments in WT compared to the allele we used in Fig 2C-D. We therefore think that NUC-1::RFP in that allele is too over-expressed and might be leaky to late endosomes. This would mean that the highly reduced lysosomal compartment numbers in the *hlh-30* mutant reported in Fig 2C-D might result from reduced late endosome numbers. We replaced the WT versus *hlh-30* mutant lysosome counts with an allele that is representative of our other NUC-1::RFP alleles (Fig 2F).

- Condense the discussion in lines 238-248 about age-related changes in lysosomal compartment size for wild-type and hlh-30 mutants, as the differences are minor and not statistically significant.

We repeated the experiment with a higher resolution, and perform the statistical analysis using a linear mixed model. With the new result, we saw a difference in lysosomal compartment size between WT and hlh-30 mutant at Day 3 and Day 6 (Fig 2G). Surprisingly, whereas our originally submitted manuscript reported no statistically significant difference in lysosomal compartment size with a trend for increased size in the *hlh-30* mutant, we now show that lysosomes in the *hlh-30* mutant are, in fact, smaller compared with WT at adult Day 3 and 6. We also observed the same trend of reduced lysosome size under ConcA treatment. In WT, we observed an increase in lysosomal compartment size between adult Day 1 and Day 3, driven by an increase in the size of the largest lysosomal compartments, but there is no additional increase with aging. We also analyzed the total lysosome volume within each neuron cell body and found similar trend. All together, we find no evidence of lysosome swelling / enlargement as a pathology of lysosome dysfunction.

- In Fig. 2E, hlh-30 mutants are missing from analysis on day 9 and day 11. Provide an explanation for this.

We added Day 9 and Day 11 mainly for comparing Day 9 WT and Day 6 *hlh-30* mutant, which suggest that the *hlh-30* mutant showed an early-onset of lysosomal acidity disruption. We made a new figure for WT Day 1 - Day 11 (Fig 1C), and only plot Day 1-Day 6 result for comparison between WT and *hlh-30* mutant (Fig 1B).

- Replace all instances of "hlh-30(o)" in the manuscript with the full allele name "hlh-30(tm1978)" or refer to it as "hlh-30 deletion." Update all figures to use "hlh-30" for clarity and adherence to standard notation.

We added this explanation of the tm1978 allele in the initial description of hlh-30 in the results section (lines 199 - 201): “The *hlh-30(tm1978)* allele is a frameshifting deletion and putative null allele. The *hlh-30(tm1978)* mutant, hereafter referred to as the *hlh-30* mutant, is viable and displays WT animal morphology and behavior.” We replaced “hlh-30(o)” with “the *hlh-30* mutant” throughout the manuscript.

- Validate the SNG-1::ARGO GFP/RFP tool using ConcA as a control experiment to confirm its reliability in detecting lysosomal changes. Include this validation in Fig. 1. Once validated, use the tool in Fig. 2 to characterize lysosomal dynamics in wild-type and hlh-30 mutants.

We were very interested in doing this experiment. Our analyses using the SNG-1::ARGO method indicate that SNG-1::ARGO is sorted for degradation at the presynapses into compartments with reduced pH – likely endolysosomes or autophagosomes – and then transported to the neuron cell body for degradation (Shiliaev et al., 2024). This sorting for degradation at the presynapses can be detected by the presynaptic SNG-1::ARGP GFP/RFP ratio, which is lower than that of fully unquenched GFP/RFP in Day 2 adult animals (Shiliaev et al., 2024). Injecting Day 3 adult animals with Bafilomycin A, another V-ATPase inhibitor similar to Concanamycin A, results in an increased presynaptic SNG-1::ARGO GFP/RFP ratio within a few hours (see panel A below, data is from Shiliaev et al., 2024).

For the suggested experiment, not only lysosomal degradation in the cell body but also autophagosome and endolysosome maturation and retrograde trafficking are expected to be inhibited by blocking the V-ATPase with chronic exposure to concanamycin A. We therefore would predict to observe an increased SNG-1::ARGO GFP/RFP ratio at both the presynapses and in the neuron cell body with steady-state imaging compared to control animals, and we would also expect to see a slower SNG-1::ARGO turnover rate from the presynapses using the pulse-chase quantification of fluorescence. The presynaptic steady-state SNG-1::ARGO GFP/RFP ratio provides a built-in means to assess whether the Concanamycin A is working: if the neuron is experiencing an appreciable dose of ConcA, we should observe a higher presynaptic SNG-1::ARGO GFP/RFP ratio in the ConcA treated animals compared to controls.

We tried the experiment several times using the same ConcA treatment protocol that we used for the other ConcA experiments in this manuscript, but unfortunately, we could not detect a change in SNG-1::ARGO GFP/RFP at the presynapses (panel B below) or in the neuron cell body (panels D and E). This indicates that the neuron is not experiencing the ConcA. Unsurprisingly based on this lack of validation that drug treatment is taking effect, the concA treated animals also show no change in SNG-1::ARGO turnover rate in the pulse-chase analysis (panel C).

These results indicate that the DA9 neurons are less sensitive to ConcA treatment. This might be due to the worm’s anatomy: the PVD neuron is directly apposed to the hypodermis, whereas the DA9 neuron is in the ventral nerve cord, surrounded by other neurons and additional layers of extracellular matrix. Or, perhaps the DA9 experiences the same concentration of the drug but is more resilient in counteracting its effects. Either way, it seems that this experiment is not workable with the current approach. It is known that putting ConcA in plates, the way we do with our PVD exposure experiments, is not a very effective way to dose animals with the drug due to the cuticle. It is more common to inject the drug (eg., Chang et al., 2017 doi: 10.7554/eLife.18459.)

Injecting animals is not feasible for the SNG-1::ARGO experiments, however. It might be possible to execute this experiment using the chemical hypersensitive *bus-17* mutant to validate the SNG-1::ARGO tool. We would try it if the reviewers feel this is a critical experiment.

Set-point HLH-30 activity in neurons operates without nuclear enrichment and declines in aging

- Clarify whether "set-point" refers to basal activity.

We replaced the “set-point activity” with “basal activity throughout the manuscript.

- The authors do not accurately describe the subcellular localization of C. elegans HLH-30 under non-stress conditions. Previously published data indicate that neither the endogenously GFP-tagged HLH-30 (PMID: 33314217, PMID: 39059513), the commonly used HLH-30::GFP overexpression strain (PMID: 28355222, PMID: 27001890, PMID: 23925298, and others), nor the neuron-specific HLH-30::GFP driven by the rab-3p promoter (PMID: 36419219) exhibit nuclear exclusion under basal conditions. This needs to be corrected throughout the manuscript. Notably, the current study indicates that HLH-30, when specifically expressed in PVD neurons, is excluded from the nucleus under basal conditions. This suggests the involvement of additional regulatory mechanisms unique to neurons. The authors should explicitly distinguish their newly developed HLH-30 strain from previously published strains and revise the manuscript. Furthermore, the authors should consider the possibility that, since HLH-30 is excluded from the nucleus in PVD cells, it could have a non-transcriptional/cytoplasmic role in promoting lysosomal function and neuronal health (e.g., TFEB can play a non-transcriptional role in mitochondrial regulation; see PMID: 3826332).

We checked all the papers suggested by the reviewer, and they either showed nuclear exclusion (which the authors referred to as cytoplasmic localization) under basal conditions (PMID: 33314217 Fig. 7C-D; PMID: 39059513 Fig. 1C), or showed low nuclear localization under basal conditions (PMID: 28355222 Fig. 6B; PMID: 27001890 Fig. 3B; PMID: 23925298 Fig. 4A). In PMID: 33314217 Fig. 7 C we can see HLH-30::GFP largely excluded from the nucleus. In PMID: 39059513 Fig. 2F and from the description of that figure we can tell the cytoplasmic localization is indeed largely excluding from the nucleus. In PMID: 28355222, we didn’t find images of HLH-30 localization, but their results do not contradict the notion that HLH-30 is largely excluding nucleus under basal conditions. And in PMID: 27001890, PMID: 36419219, PMID: 23925298 it’s hard to tell the nucleus localization with their representative images.

PMID: 33314217 Fig. 7D

Part of PMID: 33314217 Fig. 7C White arrow head is pointing another nuclei on the right corner. HLH-30::GFP in the cell body in the red box is avoiding nucleus.

Part of PMID: 39059513 Fig. 1C (representative image is too blurry to tell localization within cells)

Part of PMID: 39059513 Fig. 2F, the authors described and quantified these with cytoplasmic distribution

PMID: 28355222 Fig. 6B EV (empty vesicle) is basal condition

 PMID: 27001890 Fig. 3B

Part of PMID: 23925298 Fig. 4A.

We added this additional sentence to our description of the localization: “HLH-30::GFP11 is not completely absent from the nucleus, though, so it can still be affecting some transcriptional regulation.” (line 396 – 398).

- In lines 106-108 of the introduction, the authors state that "Additionally, HLH-30-regulated gene expression shows a declining trend with age, suggesting that reduced HLH-30 activity contributes to age-associated lysosomal dysfunction." This claim is revisited in lines 358-373 and is based on their analysis of previously published whole-body RNA-seq data, which shows decreased expression of hlh-30 and one of its target genes, rab-7. However, single-cell RNA-seq (PMID: 37531250) and single nuclei RNA-seq (PMID: 38816550) data generated from C. elegans throughout the adult lifespan are available, and the data from PMID: 37531250 specifically indicate that hlh-30 expression increases with age, including in neurons. These findings conflict with the conclusions made in this manuscript and suggest that whole-body RNA-seq data may not accurately represent gene expression dynamics in specific cell types. The authors must update Fig. 3 with an analysis of publicly available

single-cell and single-nuclei RNA-seq data to assess the expression of hlh-30 and its target genes with age specifically in PVD neurons, especially since they make conclusions on cell-autonomous functions of HLH-30. They should then reconsider and, if necessary, revise their claims based on these results.

We took a deeper look at PMID: 37531250 Fig. 7A which indicated *hlh-30* expression increases with age in neurons. This result is a summary of all cell types and comparing Day 1, Day 3, Day 5, Day 8, Day 11 and Day 15 worms. When we look at *hlh-30* mRNA levels specifically in PVD neurons using their website (<https://c.elegans.aging.atlas.research.calicolabs.com/embeddings> ), we found *hlh-30* is actually decreasing in mid adulthood (from Day 1 to Day 5), showing a similar trend as our whole-body RNA-seq data in Fig 4H (old Fig 3H). Our *rab-7* transcriptional reporter within the PVD neuron shows a similar trend as the whole-body RNA-seq result, while in the single cell RNA-seq data, *rab-7* mRNA level has very few reads at Day 3, and that is an important data point for our result. Therefore, we think in our case, our transcriptional reporter would be a better indicator for *hlh-30* activity than the single cell RNA-seq result. We weren’t able to analyze the mRNA level of other target genes using the single-cell RNA-seq data mainly because there is not enough reads for the those genes in PVD neurons.

PMID: 37531250 Fig. 7A

- Figure 3F: Why is there no data point for the hlh-30 mutant on day 9?

That WT day 9 data point was superfluous, and we removed it from the graph (current Fig 4F).

Loss of HLH-30 accelerates age-related decline in dendrite morphology maintenance

- The images of the dendrite morphology are beautiful and we would like to encourage the authors to include the images of Figure S2 in figure 4 to fully illustrate the age-related defects in dendrite morphology.

We added those images (current Fig 5A).

- The authors claim that HLH-30 functions cell-intrinsically to promote healthy neuronal morphology with age. However, the tissue-specific rescue experiments in Fig. 4D and E reveal that PVD- and pan-neuronal HLH-30 rescue only partially restore neuronal degeneration and sprouting phenotypes. In contrast, pan-somatic rescue restores these phenotypes to wild-type levels, indicating that cell-non-autonomous effects of HLH-30 likely contribute to neuronal health.

We added discussion that both cell-autonomous effect and non-cell-autonomous effect contribute to the maintenance of dendrite morphology (line 649 - 651), changed the wording to say that “neuron-intrinsic lysosomal dysfunction *contributes* to defective maintenance of dendrite morphology” (line 635-636) and refined the model (Fig 6).

- The authors should address the fact that HLH-30 expression levels are unknown in the rescue strains shown in Fig. 4D and E. This should be noted as a caveat in the discussion.

We added the caveat in the discussion (line 680 - 685).

- While the authors claim HLH-30 has a neuronal-specific, cell-intrinsic role in maintaining neuronal morphology with age, data from Figs. 4H and I suggest that HLH-30 knockdown in other tissues, such as the hypodermis and muscle, also worsens degeneration and sprouting phenotypes. This indicates that HLH-30 expression in non-neuronal tissues contributes to neuronal health. Experimental assessment and discussion of the tissue-specific nuances of HLH-30's role in maintaining neuronal morphology with age are lacking. The authors may consider removing the data from non-neuronal rescue strains to focus on the neuronal role of HLH-30 while exploring the cell-non-autonomous role of HLH-30 in future studies.

We added discussion on both the cell-autonomous part and the non-cell-autonomous part (lines 575 - 586).

- The efficiency of the AID system needs validation for each tissue-specific strain. Images and quantification of fluorescence intensity of HLH-30::GFP (or similar) under the corresponding tissue-specific promoters should be included in the supplementary materials. Validation is only necessary for the pan-somatic and pan-neuronal AID strains if data from other tissue-specific strains are removed.

We added representative images of pan-somatic HLH-30::GFP depletion under different tissue-specific promoter drived TIR1, indicating that the tissue-specific promoter drived TIR1 is depleting HLH-30::GFP in the specific tissue, and not leaky to other tissue (S3A Fig). We also tested and quantified the reduction of HLH-30::AID::GFP11 specifically in PVD neuron using pan-neuronal TIR1, suggesting that pan-neuronal TIR1 is able to deplete HLH-30 in PVD neurons (S3B-C Figs).

- Day 1 controls should be included in the graphs for Fig. 4B and C to provide a clearer baseline for age-related changes.

We added Day 1 and Day 3 PVD neuron morphology (Figs 5B and 5C).

- In lines 178 and 456-460, the authors state: "The hlh-30(o) mutant is reported to have a wild-type lifespan at 20°C and a slightly shortened lifespan at 25°C [19,74]. Importantly, the shortened lifespan at 25°C cannot be rescued with neuron-specific HLH-30 expression [74]. Therefore, the rescue of the hlh-30(o) mutant's dendrite degeneration and sprouting by PVD-specific or pan-neuronal hlh-30 expression is not an indirect effect of lifespan differences between the strains." However, other studies (PMID: 32302543 and PMID: 28198373) have reported a decreased lifespan for hlh-30(tm1978) at 20°C. The authors should measure the lifespan of hlh-30(tm1978) mutants under their experimental conditions to strengthen their conclusions and hypotheses.

We added a lifespan assay of WT vs. *hlh-30(tm1978)* at 20C (S2B Fig). The median lifespan of WT and hlh-30 mutant are both 14 days.

- The authors need to further explain the normal function and relevance of cup-5 and the nature of the cup-5(lf) mutation. Currently, the description is limited to the statement: "We also assessed the PVD dendrite morphology in cup-5(lf) mutant animals, which are known to have disrupted lysosomal functions" (lines 423-425). Additional context regarding the molecular role of cup-5 and its mutation's impact on lysosomal function would clarify its significance in the study.

We added the description (lines 512 - 514).

Minor comments:

- Suggestion to use color for fluorescent microscopy images for visual clarity (Figs. 1A and D, 2A and C, 3A and E).

We used color for merged images for visual clarity. We prefer to display single channel images in the gray scale for consistency and for reducing the messiness for PVD dendrite images in Fig 5A (original Fig 4A).

- Fig S1 is not useful without transgene names.

We added transgene names for S1 Figs.

- Fig. 2A: labeling of image 3 (hlh-30(o)_PVD>HLH-30::GFP) is not reader-friendly. Something as follows would be better: hlh-30(-) + HLH-30::GFP rescue in PVD.

We changed the labeling (current Fig 2A and 2C).

- In Fig. 3D, include a heading or other additional labeling to indicate what gene the figure is referencing. Font size could also be significantly reduced.

We added rab-7 and reduced the font size for that figure (current Fig 4D).

- Indicate day of adulthood of assayed worms within the figure for Figs. 4D-K.

We added the age for those figures (current Figs 5D-K).

- Figs. 4K and J are not referenced in order in the text (lines 413-414).

We reference those images in order in the text (line 502 - 503).

- Many graphs would benefit from more detailed y-axis labeling (similar to Fig. 2B) or headings to improve readability. E.g., Fig. 2H, I - GFP/RFP ratio of what?; Fig. 4B-K - percentage of worms with what?

We changed the labeling of original Fig 2H-I (current Fig 3I-J) and in original Fig 4 we used a color code that figures that has blue axis represent percentage of worms with dendrite degeneration, and figures with red axis represent percentage of worms with dendrite sprouting (current Figs 5B-M).

- Authors should explain why they chose to assay worms on specific days of adulthood in the manuscript text, especially since days do not match across figures (e.g., days 1 and 3 in Fig. 2G; days 3, 6, 9 and 11 in Fig. 1; days 2 and 7 in Figs. 2H and I…).

We added more data points for most figures in Fig 1 and Fig 2, and we used Day 1, Day 3, Day 6 and Day 9 for all experiments in PVD neurons. For DA9 neuron, we chose another set of timepoints following the method from another paper from our lab, so we use Day 2, Day 3, Day 4, and Day 7 for all experiments in DA9 neurons (Shiliaev et al., 2024 doi: <https://doi.org/10.1101/2024.11.26.625560>).

- Keep graph labeling font consistent. Do not mix bold and plain text (e.g., Fig. 2B x-axis).

The hlh-30(o) is italicized and looked plain. We changed it back and keep it consistent.

- Please indicate what figures each strain was used for in the strain list (Table 1).

We added that in Table 1.

Rev. 3:

This report demonstrates the role of the autophagy-related transcription factor hlh-30 in protecting neurons against the stress of aging. The authors developed interesting tools to define lysosomal function during the aging of C. elegans and show convincingly the negative effect of the loss of hlh-30 on neuronal health. The quality of the images of some of the reporters could be improved, which should address some of the issues with organelle quantification. In a few parts, some of the imaging is simplified to data points whereas representative images would remain informative. Unlike an increasing number of publications nowadays, the authors have done a thorough job of citing proper literature. Kudos to the authors, this is a well-written manuscript and an interesting study. Therefore, I recommend only a few minor revisions.

Minor comments:

In Fig. 1A and 1D, the red signal looks quite saturated and may have rendered the quantification more difficult. Possibly, lower exposure on confocal microscopy would be more suitable for quantification of small clusters of lysosomes, as noted on line 157-158. This is a relatively common issue with autophagy reporter as well that can be resolved by lower exposure.

Over-exposure seems to be an issue for the colocalization picture of NUC-1::RFP with SNT-1::GFP.

Figs 1A and 1D are actually not over-exposed during acquisition. Our confocal microscope is equipped with a CMOS camera, and our highest fluorescence intensity values for any of our fluorescence-tagged alleles tends to be on the order of 10,000 AU out of the possible 65,535. It was because of the LUT setting we applied made it looks like over-exposed. We also took images with super resolution confocal microscopy to be more accurate on the lysosome number and size counts (Figs 2D-H).

In Fig. 2D-L, the corresponding representative images for each condition should be made available in supplemental to help readers appreciate the difference (or lack thereof).

We added representative images for all conditions of ARGO experiments (current Figs 3B, 3H, and S2D-E Figs). Since the GFP/RFP ration change is not obvious looking by eye, we added a GFP/RFP ratio heat map in order to intuitively show the changes. We also added representative images for lysosome number and size counts with super resolution under different conditions (current Figs 2D-E).

A schematic of the ARGO method could help readers better understand the approach.

We added a schematic of the ARGO method (current Fig 3A).

The model, Figure 4L (note on line 476) is missing.

We applied some changes to the model and refined our model (Fig. 6).
