# Supplementary material for: Expansion of lysosomal capacity in early adult neurons driven by TFEB/HLH-30 protects dendrite maintenance during aging in Caenorhabditis elegans
Source: PLoS Biol. 2025 Sep 30;23(9):e3002957. doi: 10.1371/journal.pbio.3002957 (PMC12510649; doi:10.1371/journal.pbio.3002957)
Supplement: Attachment — Submitted filename: Zhong et al Response to Reviewers2.docx [file pbio.3002957.s012.docx]

Reviewers' comments

Rev. 1:

The authors have fully addressed my comments.

Rev. 2:

The authors have put substantial effort into revising the experimental work, but the interpretive and narrative update is incomplete. New data were added but not sufficiently integrated into the overall story. Most notably, the use of Concanamycin A (ConcA) as a pharmacological tool is not rigorously validated. Despite relying on ConcA to support their claims about lysosomal dysfunction, the authors acknowledge multiple times that it produces limited or no effect in their assays and offer speculative explanations, such as poor cuticle permeability or neuron-specific resistance, without experimentally addressing these limitations. A proper validation would require a time-course analysis, multiple doses, positive controls (e.g., injected ConcA), or use of sensitized mutant backgrounds known to enhance drug uptake. Instead, the manuscript includes only superficial treatment of these issues, and yet still draws conclusions based on the assumption that ConcA is functioning as expected. This is especially problematic in light of the failed effects in key reporter assays such as SNG-1::ARGO. In addition, the authors briefly mention published single-nucleus RNA-seq data that do not support their claim of age-related declines in lysosomal gene expression but do not explore or visualize this discrepancy. This undermines the strength of their conclusions and misses an opportunity for rigorous comparison.

We decided that the most direct validation of ConcA would be to inject it into the pseudocoelom and assess its acute impact on the fluorescence of the lysosome acidity reporter and the SNG-1::ARGO.

For the lysosome acidity reporter, worms injected with ConcA and imaged three hours later showed a significant increase of GFP/RFP ratio compared to “mock” injections. This is the expected result, as de-acidifying the lysosomal compartments through acute inhibition of the V-ATPase should diminish the quenching of GFP. We added these data to Fig. 1C. Comparing these data to the long-term, low dose of ConcA in the Nematode Growth Media, wherein we do not see the increased GFP/RFP ratio until Day 6 of adulthood, indicates that ConcA is able to affect lysosome acidity in PVD neurons at Day 3, but it takes time to go through the worms’ cuticles.

To use ConcA to validate the SNG-1::ARGO in the DA9 neuron, we used the same experimental setup, wherein we injected ConcA versus mock directly into the pseudocoelom of Day 3 worms, then imaged them 3 hours post-injection. Worms injected with ConcA showed a significantly increased SNG-1::ARGO GFP/RFP ratio compared to mock injected animals both in the neuron cell body and at the presynapse. These results provide support for the interpretations of SNG-1::ARGO fluorescence we had already put forward in the manuscript: A) the lower SNG-1::ARGO GFP/RFP ratio in the cell body versus the presynapse indicates that SNG-1 gets degraded within lysosomal compartments in the neuron cell body; and B) the SNG-1::ARGO GFP/RFP ratio at the presynapse reflects not only SNG-1 on the synaptic vesicles, where the GFP is not quenched at all, but also SNG-1 that has been sorted for degradation into acidified compartments, where the GFP is partially quenched. In addition, these results support our interpretation of our previous, unsuccessful attempts to manipulate SNG-1::ARGO fluorescence with long-term, low-dose ConcA in the nematode growth media (those data were included in our first Response to Reviewers): ConcA is able to affect lysosome acidity and presynaptic sorting within DA9 neurons in Day 3 adults, but it does not effectively pass through the cuticle and other tissues to access the DA9 neuron.

We added these new results to Fig 3 G-H.

Unfortunately, it would be hard to keep the worms alive till Day 7 post-ConcA injection, and it would also be unfeasible to inject enough animals to attempt a turnover experiment (the full turnover experiment shown in figure 3 used 280 animals, and we can inject about 20 animals per hour). Because of these challenges, it seems that the turnover experiment is not workable with ConcA injections.

We tried a few additional validation experiments as well. As described below, in our opinion, these experiments did not provide useful results, and so we did not add them to the manuscript.

- For validating ConcA as a pharmacological tool with the lysosome acidity reporter, we measured lysosome acidity in young-adults (Day 3) in *bus-17* mutant background, which is known to have disrupted cuticle integrity and so is more permeable to some drugs. However, similar to our experiments with WT animals (Fig. 1B), there was no difference between Ctrl and ConcA treatment at Day 3, but the ConcA-treated animals showed increased lysosomal GFP/RFP by Day 6. Considering this result together with the result of the ConcA injection experiment described above, it seems that the *bus-17* mutation does not sufficiently increase the permeability of the cuticle to ConcA.

- We also tested whether reducing function of another *bus* gene to increase the permeability of the cuticle would enable us to perform a SNG-1::ARGO turnover experiment with ConcA. The *bus-17* gene is tightly linked with the *heSi160* allele in that strain, so it would take substantially longer than a month to generate the *carIs1; sng-1(syb3140car2) bus17 heSi160* strain and then perform the experiment. Instead, we treated the worm with *bus-18* RNAi, which is also hypersensitive to some drug treatments. We performed the *bus-18* RNAi and *unc-22* RNAi together, as *unc-22* is a positive control for RNAi efficacy. Animals treated with *unc-22* RNAi showed the expected locomotion defects (observation, data not collected). Furthermore, the *bus-18* RNAi worms showed the expected “skiddy” locomotion phenotype and rare vulva explosions, similar to the previously described *bus-18* mutant phenotypes (observation, data not collected).

We grew the bus-18 RNAi-treated worms on ctrl vs ConcA plates from adult Day 0 (L4), as we had done for all the long-term, low-dose ConcA treatment experiments in the manuscript. Then, we performed the SNG-1::ARGO turnover experiment as described in the manuscript. Unfortunately, we did not detect a change in SNG-1::ARGO GFP/RFP in the neuron cell body. This indicates that the DA9 neuron is not experiencing ConcA treatment, so we did not continue analyzing the data we collected.

We took another look at the single nuclei RNA-seq data (PMID: 38816550), and the website for transcriptomic Cell Atlas of Worm Aging (CAWA, <http://mengwanglab.org/atlas>). This single nuclei RNA-seq data analyze WT worms at Day 1, Day 6, Day 12, and Day 14. But in our case, we are more focused on changes in mid-adulthood (Day 1 - Day 6), with Day 3 being an important time point. In addition, as described in our previous Response the Reviewers, we assessed the single-cell data from PMID: 37531250. The well-validated transcriptional targets of HLH-30 that we use in our manuscript had very few to no reads in the PVD neuron in those data. Therefore, we think in our case, our transcriptional reporter and the whole-animal transcriptomics we used would be a better indicator for *hlh-30* activity than the single nuclei RNA-seq result.

Other changes:

- We fixed a few typos and grammatical errors throughout the manuscript

- We revised Supplemental Table 2 to be more complete

- In Fig5D-E, we added the comparison between the *hlh-30* mutant and WT Day 6.
